# Randomized phase II study of stereotactic body radiotherapy and interleukin-2 versus interleukin-2 in patients with metastatic melanoma

**DOI:** 10.1136/jitc-2020-000773

**Published:** 2020-05-27

**Authors:** Brendan Curti, Marka Crittenden, Steven K Seung, Christopher B Fountain, Roxanne Payne, ShuChing Chang, Jessica Fleser, Kimberly Phillips, Ian Malkasian, Lyn B Dobrunick, Walter J Urba

**Affiliations:** 1 Providence Cancer Institute, Earle A Chiles Research Institute, Providence Portland Medical Center, Portland, Oregon, USA; 2 Division of Radiation Oncology, The Oregon Clinic, Portland, Oregon, USA; 3 Providence Cancer Institute, Providence Portland Medical Center, Portland, Oregon, USA; 4 Medical Data Research Center, Providence St Joseph Health, Portland, Oregon, USA

**Keywords:** melanoma, radiotherapy, clinical trials, phase II as topic

## Abstract

**Background:**

A pilot study of stereotactic body radiation therapy (SBRT) followed by high-dose interleukin-2 (IL-2) showed a higher than anticipated objective response rate (ORR) among patients with metastatic melanoma (MM). We performed a prospective randomized study to determine if the ORR of SBRT + IL-2 was greater than IL-2 monotherapy in patients with advanced melanoma.

**Methods:**

Patients with MM who had adequate physiological reserve for IL-2 and at least one site suitable for SBRT were eligible. There was a 1:1 randomization to SBRT + IL-2 or IL-2 monotherapy. Patients received one or two doses of SBRT (20 Gy per fraction) with the last dose administered 3 days before starting the first cycle of IL-2. IL-2 (600,000 IU per kg via intravenous bolus infusion) was given every 8 hours for a maximum of 14 doses with a second cycle after a 2-week rest. Responding patients received up to six IL-2 cycles. Patients assigned to IL-2 monotherapy who exhibited progression of melanoma after cycle 2 were allowed to crossover and receive SBRT and additional IL-2. Response Evaluation Criteria in Solid Tumors 1.1 criteria were applied to non-irradiated lesions for response assessment.

**Results:**

44 patients were included in the analysis. The ORR in the SBRT + IL-2 group was 54%: 21% complete response (CR), 33% partial response (PR), 21% stable disease (SD) and 25% progressive disease (PD). The ORR in patients receiving IL-2 monotherapy was 35%: 15% CR, 20% PR, 25% SD and 40% PD. Seven patients assigned to IL-2 subsequently received SBRT + IL-2. One CR and two PRs were observed in the crossover group. There was no difference in progression-free or overall survival (OS). At 5 years the OS was 26% in the SBRT + IL-2 group and 25% in the IL-2 monotherapy group. The disease control rate (DCR) was higher in the SBRT + IL-2 group (75% vs 60%, p=0.34).

**Conclusions:**

SBRT + IL-2 induced more objective responses with a higher DCR compared to IL-2 monotherapy in MM. IL-2 monotherapy resulted in a significantly higher ORR than anticipated. Some patients in the crossover group also achieved objective responses.

**Trial registration number:**

NCT01416831.

## Background

The first publication reporting the efficacy of high-dose (HD) interleukin-2 (IL-2) for patients with metastatic melanoma appeared in 1985; a subsequent manuscript describing 270 patients treated with HD IL-2 reported a complete response (CR) rate of 6% and a partial response (PR) rate of 10% with a median duration of response greater than 40 months.^1 2^ Over 70% of patients achieving a CR and approximately 15% of those achieving a PR were alive and without recurrence at 15 years identifying HD IL-2 as the first curative immunotherapy regimen for patients with stage IV melanoma. Since 2010 there have been many significant advances in melanoma treatment including the development of checkpoint antibodies, first anti-CTLA-4 using ipilimumab,^3^ then anti-PD-1 with nivolumab,^4^ and now the use of combined T-cell checkpoint therapy with ipilimumab and nivolumab showing an objective response of 58% and complete response of 19% associated with 3-year survival of 52%.^5^ Clinically significant responses and disease control have also been demonstrated with anti-PD-1 checkpoint monotherapy with nivolumab or pembrolizumab.^6 7^ Targeted therapy with the BRAF and MEK inhibitors vemurafenib and cobimetinib, dabrafenib and trametinib or cobimetinib and encorafenib are also associated with a high probability of objective response and improvement of disease-free and overall survival. Complete regressions with BRAF-targeted therapy are also possible and associated with improved long-term outcomes.^8^ Improved survival has been validated for T-cell checkpoint inhibitor (CPI) therapy and BRAF-targeted therapy combinations, yet the proportion of patients with complete and durable responses who require subsequent therapy based on progression-free survival probability is at least 60% and may be as high as 80% at 3 years.^5 6^ Furthermore, the best therapy or therapeutic sequence for patients who have melanoma progression after CPI or targeted therapy is not yet known and most patients with metastatic disease still die as a consequence of melanoma as illustrated by recent survival statistics.^9^

Preclinical studies indicate that exposure of tumor cells to high-dose radiation can augment the release of inflammatory cytokines, upregulate expression of MHC class I, B7.1, and Fas/CD95.^10–15^ Tumor cells injured by radiation can also release damage-associated molecular patterns (DAMPs) such as HMGB1 and double-stranded DNA (dsDNA) that can trigger a TLR4-dependent cognate immune response.^16^ High-dose per fraction radiation also increases tumor infiltrating activated CD8^+^ T cells and has been associated with enhanced tumor control at distant sites when combined with immunomodulatory agents in preclinical studies.^17–19^

We observed that patients with melanoma or renal cell carcinoma (RCC) who had radiation for urgent palliation in the week before IL-2 had a surprisingly high objective response in lesions that were not radiated following high-dose IL-2. This observation led us to perform a pilot phase I trial of stereotactic body radiation therapy (SBRT) and IL-2 in which the primary objective was to determine the maximum tolerated dose (MTD) of SBRT. We observed an objective response of 71% in previously untreated patients with metastatic melanoma and 60% in RCC. There was no increase in the toxicities associated with high-dose IL-2 and no dose-limiting toxicities associated with radiation. These encouraging initial clinical signals were the basis for the phase II trial reported here.

The primary clinical aim of the phase II study was to compare the objective response of SBRT + IL-2 versus IL-2 monotherapy in the non-irradiated lesions. Secondary objectives included an evaluation of crossover to SBRT + IL-2 in patients who progressed on IL-2, and an investigation of DAMPs, which as stated above may be important immune adjuvants that influence the response to immunotherapy and radiation. Due to the technical complexities of measuring HMGB-1 dsDNA in the peripheral blood, we chose instead to measure surrogate markers for DAMPs including uric acid, lactate dehydrogenase (LDH) and phosphorus, which reflect cell and DNA damage after radiation and procalcitonin, which reflects tissue injury and cytokine induction.

This study started in late 2011, which was the time that T-cell checkpoint antibodies and BRAF-targeted therapy were entering clinical practice. The availability of these melanoma treatment options significantly influenced accrual to this clinical trial and the clinical histories of the study population as detailed below.

## Methods

### Study design and population

A single institution phase II study was conducted at the Earle A. Chiles Research Institute, Providence Portland Medical Center (PPMC), Providence Cancer Institute. The main eligibility criteria were patients >18 years old, Eastern Cooperative Oncology Group (ECOG) performance status 0 to 1, histological confirmation of melanoma, at least one metastatic lesion amenable to SBRT in the lung, mediastinum or liver, and at least one other metastatic site not treated with SBRT. Cardiopulmonary status sufficient to tolerate HD IL-2 was required^20^ and patients had essentially normal hematologic, hepatic, and renal function before treatment. Exclusion criteria included having no metastatic site amenable to SBRT; active infection; previous radiation to sites proposed for SBRT; need for chronic steroids or active autoimmune disease. Signed informed consent was obtained prior to enrollment. The study was listed on Cancer.gov. There were no restrictions for prior melanoma systemic therapies or for brain metastases as long as they were stable or improved after local therapy (surgery and/or stereotactic radiation).

### Procedures

SBRT planning was performed using a four-dimensional CT scan with the patient in the treatment position immobilized with the BodyFIX (Elekta, Atlanta, Georgia). The internal target volume was delineated on the planning CT and a 3 to 5 mm planning target volume margin was used. An intensity modulated radiation therapy treatment plan with 6 MV photons was generated using Pinnacle V.9.0 software (Philips Medical Systems, Andover, Massachusetts) based on tumor location and geometry. The target was localized with cone beam CT before each treatment, which was delivered on the Synergy S (Elekta, Atlanta, Georgia) machine. A minimum of one and a maximum of three lesions were treated with SBRT. Clinical criteria were used to select the lesion(s) to be treated with radiation. Lesions that were causing pain or compressing a hollow viscus (such as bronchus or bile duct) were prioritized over others. In patients where no symptoms were being caused by their metastatic sites, then the lesion deemed safest to administer radiation was selected. A maximum diameter of 7 cm was allowed for each SBRT target lesion. All patients were treated by two of the authors (SKS and MC). The first eight patients were treated with one SBRT dose on the Friday before the Monday on which IL-2 was to start. The subsequent 16 patients were treated with two SBRT doses to the target lesion on the Wednesday and Friday before the Monday IL-2 start. The protocol was modified due to the observation that a melanoma patient treated with one SBRT dose had progression of the radiated lesion.

Patients who signed informed consent were assigned to treatment group in a 1:1 proportion using randomization by the closed envelope method. Of the 50 patients who signed consent, six were excluded (three due to rapid melanoma progression during screening, two due to cardiac ischemia on exercise tolerance testing and one due to insurance issues).

Interleukin-2 (Prometheus Pharmaceuticals, Dallas, Texas) treatment began on the Monday following the last radiation treatment and was administered at 600,000 IU per kilogram intravenousbolus infusion every 8 hours × 14 planned doses with an additional cycle given after a 16-day hiatus (two cycles=one course of IL-2). Imaging was obtained after each course and patients with tumor regression could receive up to three courses. After IL-2 was completed, imaging was obtained every 3 months until progression or 24 months of follow-up. If patients did not have progression at 24 months, then imaging was obtained every 4 months through 3 years after the start of IL-2 and then every 6 months in year 4 and thereafter. Patients assigned to the IL-2 monotherapy group and who showed progression of melanoma after course 1 could receive SBRT and additional IL-2 cycles contingent on response. We employed PPMC Biotherapy Program guidelines for IL-2 management, which are a modification of published IL-2 dosing rules.^20 21^

This protocol used a modified version of the Response Evaluation Criteria in Solid Tumors (RECIST) V.1.1.^22^ The overall response assessment included all measurable and non-measurable target lesions except the lesions treated by SBRT, which were assessed separately. Both CT and positron emission tomography imaging were employed to assess response. A further modification of RECIST criteria was used to assess tumor bulk as reported in online supplementary figure 3 (absolute spider plots). The sum of the long axis diameter of the largest 30 tumor deposits was added and compared over time.

10.1136/jitc-2020-000773.supp1 Supplementary data

Markers of tumor lysis, inflammation and immune activation were explored by measuring serum lactate dehydrogenase, procalcitonin, uric acid and phosphate as surrogate markers of cytotoxicity-mediated antigen release with measurements obtained at baseline, after radiation but before IL-2 and after IL-2 was completed in cycle 1.

### Outcomes

The primary study endpoint was to determine the best overall tumor response rate of high dose IL-2 versus SBRT + high-dose IL-2 using RECIST criteria applied to all target and non-target lesions with the exclusion of sites treated with SBRT. We assumed in designing the study that the objective response to IL-2 monotherapy would be 16% based on other published data^23^ while the response to SBRT + IL-2 would be 60% based on our initial pilot study.^24^ Using the Pearson χ^2^ test with continuity correction, enrolling 22 patients per group (44 total) would have an 80% power to detect a difference using the response assumptions detailed above. For patients who received SBRT after progression on IL-2 monotherapy, the response rate was recorded, but not counted in the assessment of overall tumor response for the primary objective. The secondary objectives were hypothesis generating and were not included in determining the sample size.

### Statistical analysis

Baseline characteristics were compared using Fisher’s exact tests and Wilcoxon rank-sum test for continuous and categorical variables, respectively. The endpoint for overall survival (OS) was the time from the date treatment started to death. The endpoint for progression-free survival (PFS) was the time between the start of IL-2 treatment to the event related to the disease (progressive disease (PD) after initial CR, PR or stable disease (SD)) or death. To examine whether there was a difference in OS and PFS between two treatment groups, Kaplan-Meier and Cox proportional-hazards regression analyses were performed. The association of age, gender, LDH, BRAF status with OS and PFS were also evaluated. In addition, logistic regression analyses were employed to determine the independent predictors of disease control rate (DCR) (CR/PR/SD). All statistical analyses were performed using R V.3.5.0 (R Core Team, 2018).

## Results

### Population

Forty-four eligible patients with advanced melanoma enrolled and were treated from August 2011 through March 2017. Twenty-four patients were randomized to receive SBRT + IL-2 and 20 patients were assigned to IL-2 monotherapy. Of the eight patients randomized to IL-2 whose melanoma progressed after the first imaging, seven agreed to participate in the crossover portion of the study. Five additional patients signed consent but withdrew consent before receiving treatment as they opted for other systemic therapies and were not included in this analysis. Table 1 summarizes clinical characteristics and treatments before and after IL-2. There were no statistically significant differences in demographic characteristics, or baseline tumor burden, although patients assigned to the SBRT + IL-2 group tended to have a greater tumor burden compared with the IL-2 monotherapy group (online supplementary table 1). The mean LDH among patients assigned to SBRT + IL-2 was 389 compared with 263 IU/L in patients assigned to IL-2 monotherapy (upper limit of normal 268 IU/L). A higher proportion of patients in the IL-2 monotherapy cohort had melanomas with a BRAF V600E mutation compared with those assigned to SBRT + IL-2 (55% vs 29%, p=0.1).

10.1136/jitc-2020-000773.supp2 Supplementary data

**Table 1:** Clinical characteristics and treatments of study participants by group

	SBRT + IL-2	IL-2
Male	18	16
Female	6	4
Age (mean)	53	57.5
BRAF status		
Mutated/wild type/unknown	7/14/3	11/5/4
Baseline LDH (mean) (upper limit of normal 268 IU/L)	389	263
Prior therapy		
Surgery (%)	24 (100)	20 (100)
Radiation (n)	1 (4)	3 (15)
BRAF therapy (n)	1	0
Immune checkpoint (n)	5	3
Subsequent therapy		
BRAF therapy (n)	4	5
Immune checkpoint (n)	11	6
Metastatic sites at start of treatment		
Lung (%)	64	74
Liver (%)	36	37
Lymph node (%)	36	53
Bone (%)	14	5
Subcutaneous (%)	27	11
Brain (%)	5	5
Soft tissue (%)	50	21
Adrenal (%)	14	0
Sum of diameters of target lesions using modified RECIST		
cm (median)	8.2	6.5
cm (mean)	5.1	5.3
Sum of diameters of 30 largest lesions		
cm (median)	19.7	13.7
cm (mean)	11.4	10.7

LDH, lactate dehydrogenase; RECIST, Response Evaluation Criteria in Solid Tumors.

### Outcomes

**Figure 1:**
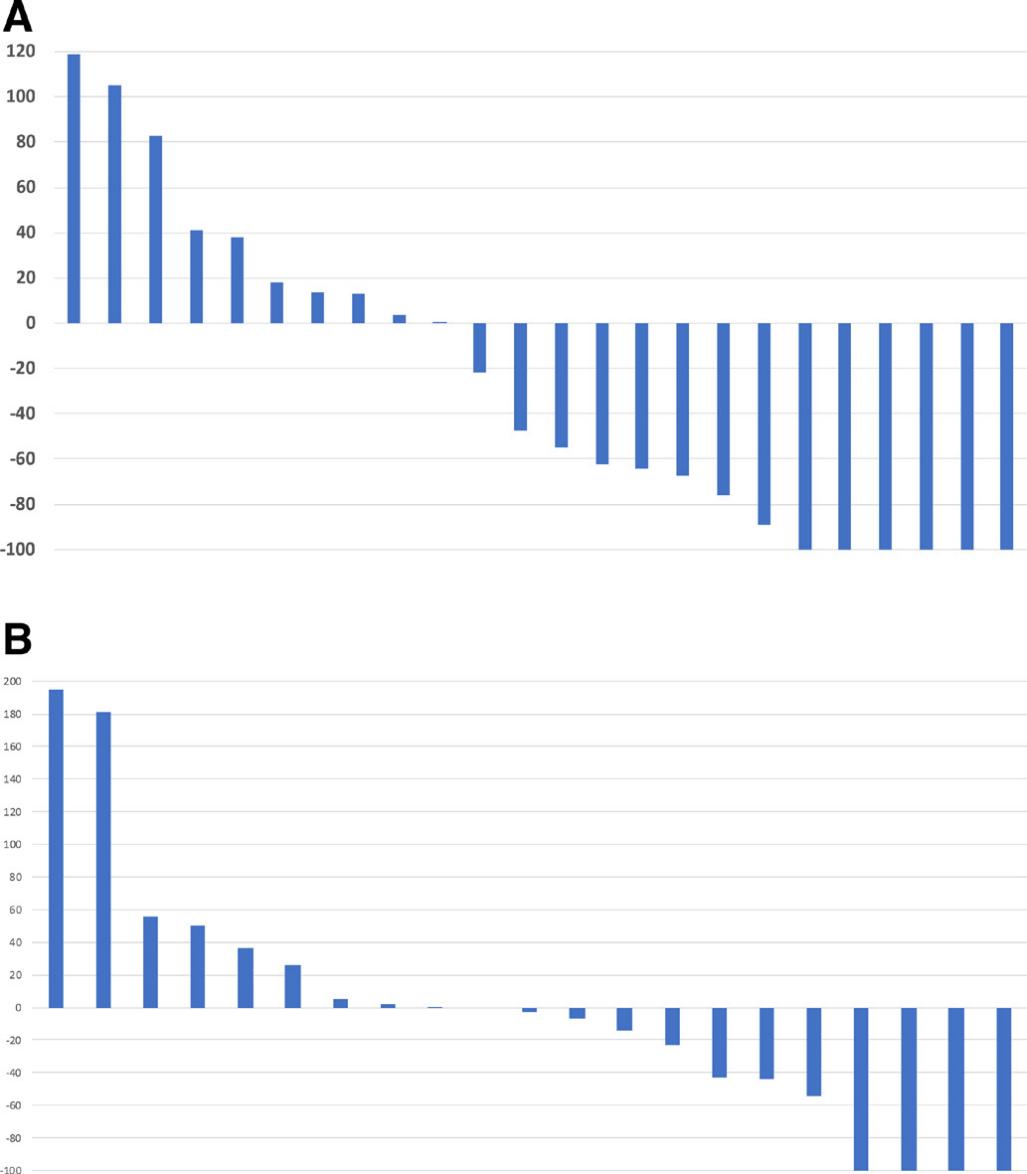
(A) Waterfall plot of best response to SBRT + IL-2. The radiated lesions were excluded from RECIST assessment of target lesions. The responses of patients who participated in the crossover portion of the study are excluded from this analysis. (B) Waterfall plot of best response, IL-2 monotherapy before crossover. The responses to additional IL-2 cycles after crossover are excluded. IL-2, interleukin-2; RECIST, Response Evaluation Criteria inSolid Tumors; SBRT, stereotactic body radiation therapy.

**Figure 2:**
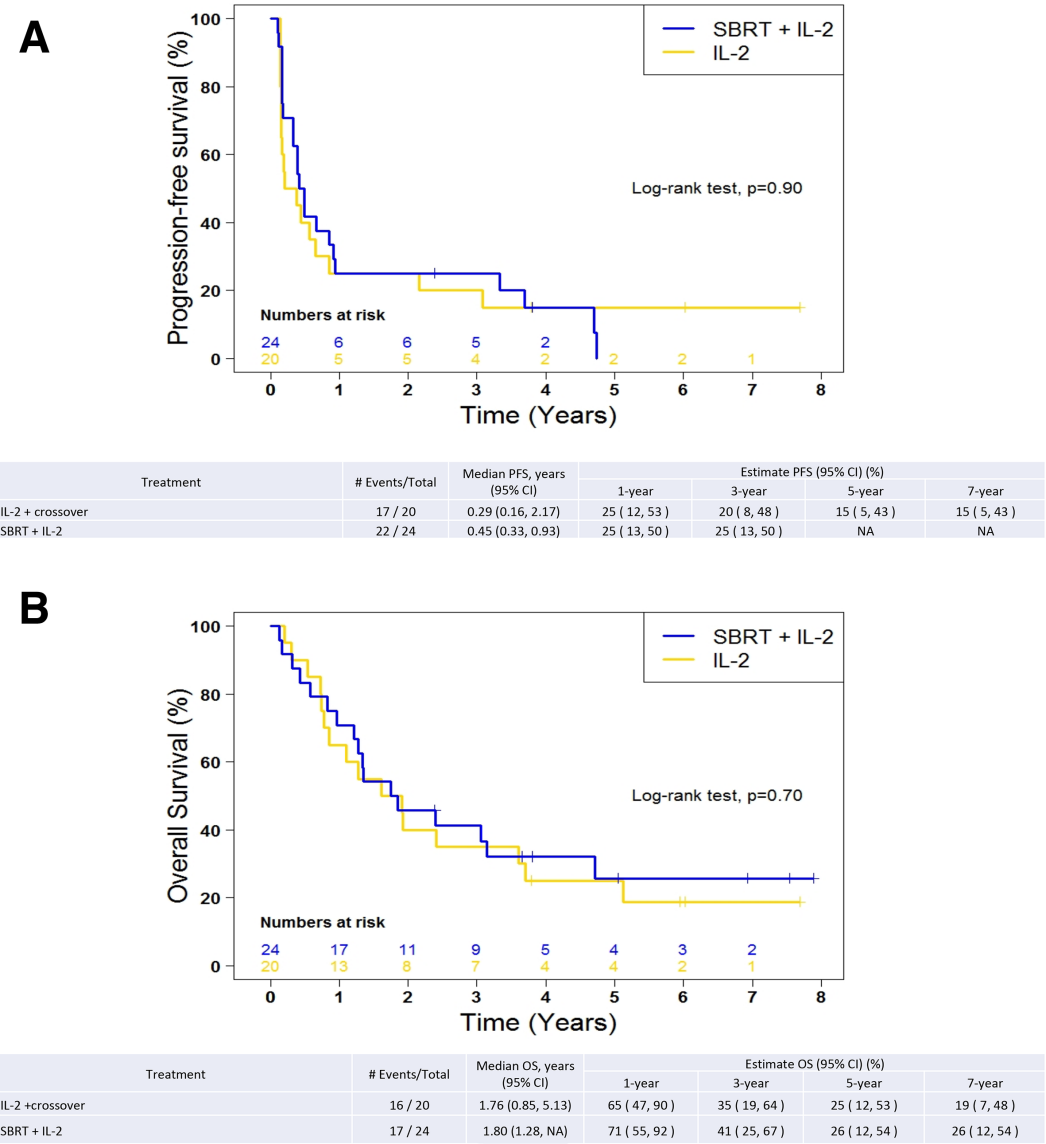
(A) Kaplan-Meier plot of progression-free survival by treatment group. Patients who participated in the crossover group are included with the IL-2 group. (B) Kaplan-Meier plot of overall survival by treatment group. Patients who participated in the crossover group are included in the IL-2 group. IL-2, interleukin-2; OS, overall survival; PFS, progression-freesurvival; SBRT, stereotactic body radiationtherapy.

Figure 1 shows waterfall plots of best overall response using RECIST 1.1 criteria after SBRT + IL-2 of the target lesions not treated with SBRT (1A) and IL-2 monotherapy (1B). The objective response in the SBRT + IL-2 group was 21% CR, 33% PR, 21% SD and 25% PD. Among the patients who received SBRT + IL-2, the best response was determined after course 1 in eight patients (33%), after course 2 in eight patients (33%) and after course 3 in eight patients (33%). Patients receiving IL-2 monotherapy had 15% CR, 20% PR, 25% SD and 40% PD. The DCR was 75% in the SBRT+IL-2 group and 60% in the IL-2 monotherapy group (p=0.34). Among the patients who received IL-2 monotherapy, the best response was determined after course 1 in 11 patients (55%), after course 2 in two patients (10%) and after course 3 in seven patients (35%). Seven patients assigned to IL-2 monotherapy participated in the crossover. One patient achieved a CR and two achieved PR of the lesions not treated with SBRT. Progression-free survival is depicted in figure 2A and overall survival in figure 2B. There was no difference in progression-free or overall survival among the treatment groups. The 1-year, 3-year and 5-year survivals were 71%, 41% and 26%, respectively, in the SBRT + IL-2 group. For the IL-2 monotherapy group (including crossover patients), the 1-year, 3-year and 5-year survivals were 65%, 35% and 25%, respectively. The 1-year, 3-year and 5-year survivals for IL-2 monotherapy excluding the crossover group were 62%, 46% and 29%, respectively.

**Figure 3:**
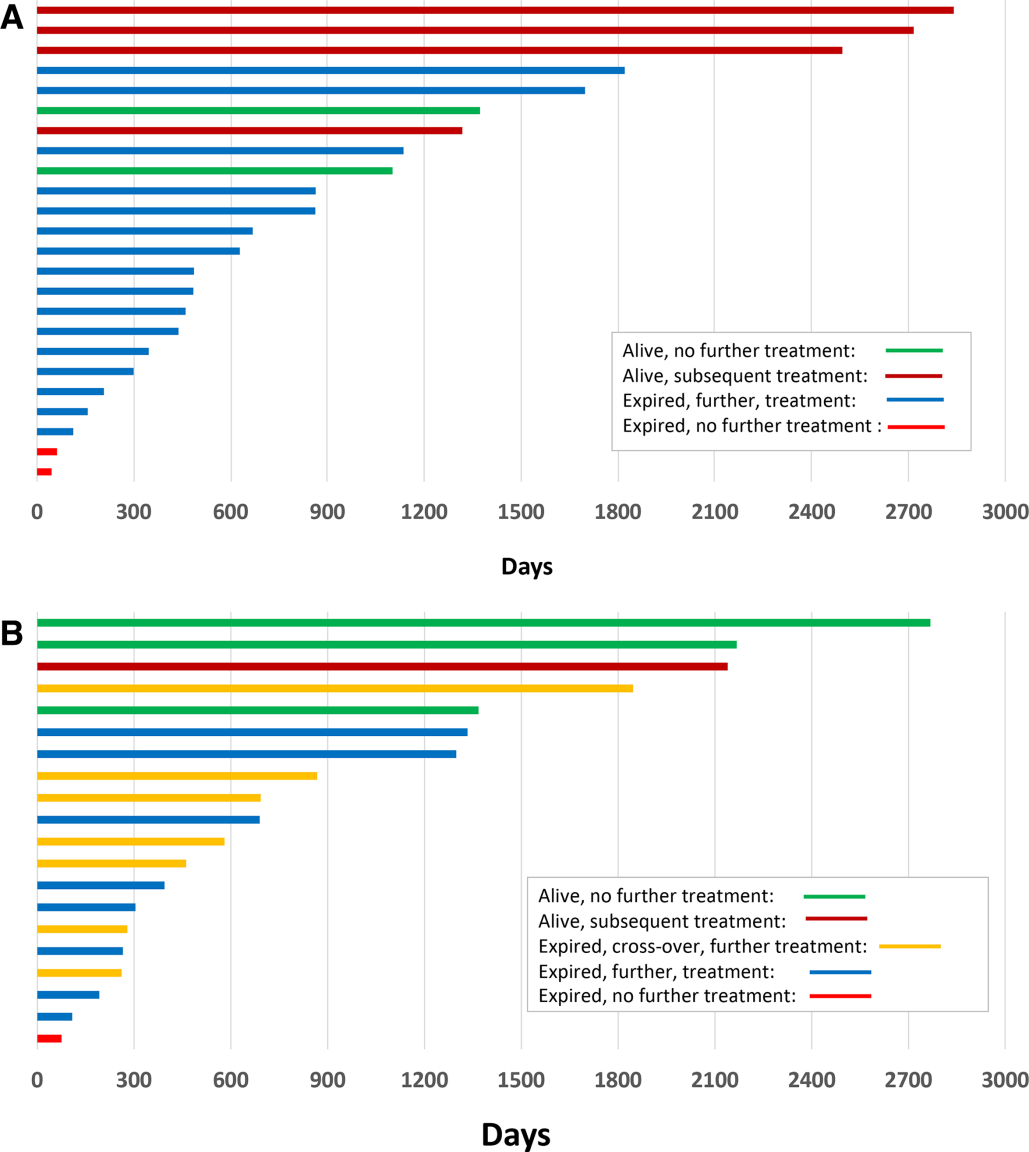
(A) Swimmers plot for SBRT + IL-2 patients. Each bar represents an individual patient’s treatment history with the bar color indicating survival status and subsequent treatment. (B) Swimmers plot for IL-2 patients (including crossover). Each bar represents an individual patient’s treatment history with the bar color indicating participation in the crossover, survival status and subsequent treatment. IL-2, interleukin-2; SBRT, stereotactic body radiation therapy.

The clinical outcomes and subsequent treatments of all 44 patients over time are summarized using swimmers plots (figure 3A for the SBRT + IL-2 group and figure 3B for IL-2 monotherapy + crossover groups). Of the 24 patients assigned to the SBRT + IL-2 cohort, four received BRAF-targeted therapy and 14 received CPI at the time of progression. This includes the 13 patients who achieved an initial CR or PR by RECIST criteria to SBRT + IL-2. Of the seven patients who achieved an initial CR or PR after IL-2 monotherapy, four required subsequent treatments. Five patients received CPI therapy before SBRT + IL-2 and three before IL-2 monotherapy. Five of the eight patients receiving CPI before enrolling on this trial had responses to IL-2, two of which were durable (lasting more than 1 year). Seven of the 18 patients who received CPI after SBRT + IL-2 had a partial regression of disease including one crossover patient. The 11 patients who did not achieve a PR with subsequent CPI had a brief interval of stability followed by progression of their melanoma. One of three patients who received IL-2 monotherapy (and no radiation) achieved a PR with subsequent CPI. Of the seven patients participated in the crossover, five received BRAF-targeted therapy and five received CPI at the time of melanoma progression. Two patients in the SBRT + IL-2 group and three patients assigned to the IL-2 monotherapy group have not required additional systemic or local treatments for their melanoma.

As described by others, patients with higher than normal baseline LDH levels had a lower probability of response (p=0.08). We also performed an analysis of the response of individual tumor lesions based on the clinical observation that many patients experienced mixed responses with regression of some lesions and progression or the development of new lesions. This analysis did not change the response interpretation as defined by RECIST. Online supplemental figures S1−S3 illustrate the tumor response in relation to the absolute tumor burden (sum of long-axis diameters of all lesions on CT).

10.1136/jitc-2020-000773.supp3 Supplementary data

10.1136/jitc-2020-000773.supp4 Supplementary data

10.1136/jitc-2020-000773.supp5 Supplementary data

### Response of lesions treated with SBRT

Patients received SBRT either as part of their initial cohort assignment before cycle 1 IL-2 or if they progressed after IL-2 monotherapy and opted to participate in the crossover group. Crossover patients received SBRT before cycle 3 of HD IL-2. When the protocol started enrolling patients, only one SBRT dose was specified as there appeared to be no difference in the outcome of patients in the completed pilot study comparing one, two or three SBRT doses before IL-2.^24^ However, among the patients assigned to a single SBRT dose we observed rapid progression of a liver lesion. The protocol was modified to administer two SBRT doses thereafter. Eight patients assigned to the SBRT + IL-2 group received one SBRT dose and 16 patients received two SBRT doses. Six of the seven patients in the crossover group received two SBRT doses. Table 2 summarizes the characteristics of the lesions treated with SBRT. The median diameter of lesions in patients assigned to the SBRT cohort was smaller compared with the patients who participated in the crossover (2.5 vs 4.5 cm, p=0.08). There was no significant difference in the organ site selection for SBRT comparing the groups. Among patients who experienced regression of tumors at the non-irradiated sites, the median size of the lesions treated with SBRT was 1.6 cm compared with 2.9 cm in patients whose lesions in non-irradiated sites did not regress. Of the 31 tumors treated in the SBRT cohort, 27 decreased in size (15 complete and 12 partial responses using RECIST criteria) and 4 increased in size. In the crossover group, nine lesions were treated of which seven decreased (three complete and four partial responses of the irradiated lesions) and two increased. We also investigated the degree and duration of responses in non-irradiated lesions in relation to the site chosen for SBRT (summarized in table 3). The study was not powered to detect differences in response based on the site selected for SBRT; however, we observed no difference in response based on 1 versus >1 site radiated or the number of radiation fractions (50% vs 56%). There was a trend favoring response of irradiating lung tumors versus liver lesions or other sites (58% vs 33%).

**Table 2:** Characteristics of the lesions treated with SBRT.

	SBRT	SBRT crossover
Median lesion size (cm)		
Baseline (range)	2.5 (0.5 to 6.4)	4.3 (1.6 to 7.1)
Assessment 1	1.3 (0 to 5.5)	2.5 (0 to 7.5)
Assessment 2	0 (0 to 6.1)	2.5 (0 to 6.3)
Median lesion # treated per patient	1	1.5
Sites treated		
Lung (%)	14 (45)	2
Liver (%)	11 (35)	4
LN (%)	4 (13)	3
Other (%)	2 (6)	0
Lesions that progressed (%)*	5 (16)	2 (22)
Lesions that regressed (%)	25 (81)	7 (78)
Lesions with no change (%)	1 (3)	0 (0)
Lesions BORR=0 (%)	15 (48)	3 (33)

#, number; BORR, best overall response rate; LN, lymph node; SBRT, stereotactic body radiation therapy.

**Table 3:** Response of non-irradiated lesions in relation to SBRT treated site

SBRT site (n)	% change of non-irradiated lesions	Median % change	Mean % change	Duration (days)	Median duration days
Liver (7)	–77, 13, –100, 4, 119, −100, 18	4	−18	289, 188, 350, 162, 57, 306, 69	188
Lung (10)	−62, 0, –100, 14, 89, 38, –23, –100, 41, 0	0	−11	767, 105, 399, 81, 342, 83, 39, 1238, 67, 169	169
Lymph node (4)	–57, –100, –100, –47	−78.5	−76	76, 1726, 690, 258	474
Bone (1)	−53	−53	−53	334	334
Liver + lung (1)	−100	−100	−100	793	793
Lung + bone (1)	−67	−67	−67	129	129

SBRT, stereotactic body radiation therapy.

**Table:** 

Crossover patients	% change	Median % change	Mean % change	Duration (days)	Median duration days
Liver (2)	–100, –100	−100	−100	157, 278	217
Lung (2)	–100, –18	−59	−41	377, 168	272
Lymph node (1)	56	56	56	56	56
Liver + lung (1)	−12	−12	−12	83	83

### Treatment received and safety

The median number of IL-2 doses for the first and second cycles of therapy were 10 and 7 in the SBRT + IL-2 group and 9 and 8 in the IL-2 monotherapy group (p=NS). This was comparable to the median number of IL-2 doses tolerated historically by other patients in our Biotherapy Program who did not receive SBRT.^21^

Anticipated toxicities from IL-2 were observed and included hypotension requiring vasopressor support, pulmonary capillary leak with hypoxemia, fever, rigors, myalgias, arthralgias, pruritus, erythematous rash, diarrhea, nausea, electrolyte abnormalities, elevations of hepatocellular enzymes, azotemia, peripheral neuropathy, mental status changes and immune-mediated hypothyroidism. These toxicities were transient (with the exception of immune-mediated hypothyroidism) and resolved using supportive medications and holding IL-2 doses. There were no long-lasting toxicities from IL-2 with the exception of hypothyroidism requiring levothyroxine in 16 patients and vitiligo in 6 patients, all of whom had regression of melanoma during treatment.

One patient assigned to the SBRT + IL-2 cohort developed respiratory failure after cycle 2 IL-2 characterized by patchy infiltrates in both lungs and outside the radiation ports in the right lateral and left anterior lung fields. He did not improve on broad-spectrum antibiotics or after diuresis, bronchodilators, empiric steroids and mechanical ventilation. Multiple cultures did not reveal an infectious etiology. Imaging revealed regression of some of the melanoma deposits in the lungs including the lesions treated with SBRT as well as other pulmonary metastatic sites. The clinical differential diagnoses included atypical pneumonia, lymphangitic spread of melanoma or immune-mediated lung injury. In light of the patients advanced melanoma, the patient opted for best supportive care and he died as a consequence of respiratory failure. The patient did not consent to invasive procedures for biopsy and his family declined to have an autopsy performed.

### Peripheral blood DAMPs

**Figure 4:**
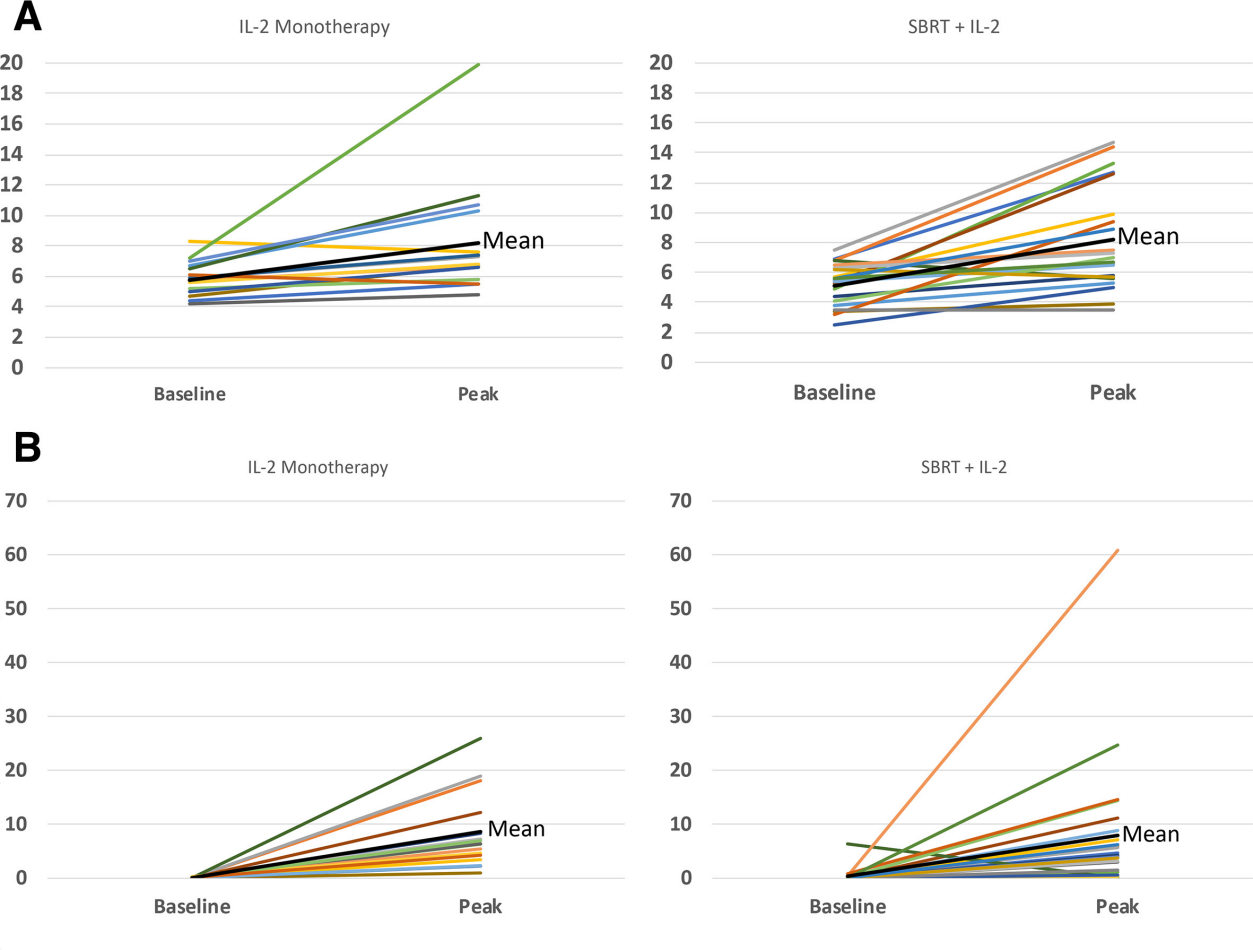
(A) Uric acid (mg/dL) by treatment group comparing baseline and peak values during cycle 1. In most patients, the peak uric acid value occurred on day 5 or 6 after IL-2 started. There was no statistically significant difference in the timing or the peak uric acid level comparing SBRT + IL-2 or IL-2 monotherapy although there was a trend toward high uric acid levels among SBRT + IL-2 responders compared with non-responders. (B) Baseline and peak procalcitonin (ng/ml) by treatment group during cycle 1. The peak procalcitonin was observed on day 5 or 6 after IL-2 started. There was no statistically significant difference in the timing or the peak procalcitonin level comparing SBRT + IL-2 or IL-2 monotherapy although there was a trend toward lower procalcitonin levels among SBRT + IL-2 responders compared with non-responders. IL-2, interleukin-2; SBRT, stereotacticbody radiation therapy.

Surrogate markers for DAMPs including uric acid, LDH, procalcitonin and phosphorus were measured serially and compared by treatment assignment. Figure 4 shows baseline and peak values for uric acid and procalcitonin. Responding patients who received SBRT displayed a trend toward higher median uric acid and lower median procalcitonin levels on days 5 and 6 after the start of IL-2 compared with patients who did not have a response to SBRT + IL-2 or those assigned to IL-2 monotherapy. There were no statistically significant changes in any of the DAMP surrogate markers measured due to patient-to-patient variation.

## Discussion

The combination of SBRT and IL-2 showed a trend toward higher response and higher DCR compared with IL-2 monotherapy in patients with advanced melanoma yet no statistically significant differences in PFS or OS were observed. We were surprised to observe the much higher than anticipated objective response of the IL-2 monotherapy arm. As expected, some patients in both arms achieved durable regressions of their melanoma and did not require further systemic or local therapy; however, the majority of patients required subsequent medical, surgical or radiation therapy to manage their melanoma. Overall, the duration of response in each group was less than anticipated based on prior reports with IL-2 monotherapy from our group and others.^21 25^ The shorter than anticipated response duration may have been due to prior therapy altering the immune response or the responsiveness of the melanoma to an IL-2-based regimen. Although overall survival could not be compared due to the crossover design, the 1-year, 3-year and 5-year survivals for patients in both arms were clinically relevant. The overall survival at 1, 3 and 5 years after CPI or BRAF-targeted therapy independent of IL-2 immunotherapy in patients with advanced melanoma is significant, yet the reality for the majority of patients is that multiple lines of systemic therapy are needed. Our long-term results are similar to more contemporary reports of patients receiving high-dose IL-2. For instance, Davar *et al*, describe 1-year, 2-year and 3-year survivals of 41%, 20% and 14%, respectively, in a large single-institution study comprising 243 patients with advanced melanoma.^26^ After progression on IL-2, 36 patients were treated with T-cell checkpoint antibodies and 7 (19%) have ongoing response to immunotherapy. The long-term survival of patients in both treatment groups in this study was probably influenced by subsequent therapy in light of the current knowledge about survival benefit of CPI and BRAF-targeted therapies. Similarly, anti-PD-1 and anti-CTLA-4 before IL-2 likely influenced IL-2 responses. Although no prospective study on clinical outcome exists for the activity of checkpoint inhibitors after IL-2, retrospective observations suggest a higher than anticipated response to anti-PD-1 following IL-2.^25^ In this report, 18% of patients achieved partial regressions of melanoma after their high-dose IL-2 regimen.

The rationale for investigating the combination of high-dose per fraction radiation and IL-2 was not based on the assumption of an abscopal effect. Rather, we hypothesized that cell death from radiation supplied a source of antigen for adaptive immune response and that the IL-2 provided a strong cytokine signal to amplify immune response.^27^ As a consequence of the radiation-induced cell death, we also expected to observe higher uric acid levels in patients who received radiation. In addition, we hypothesized that procalcitonin would be lower as the immune response would be directed to the radiated tumor and not manifest systemically as is the case with high-dose IL-2. There were no statistically significant differences among the treatment groups due to significant patient-to-patient variation, but there was a trend toward higher uric acid and lower procalcitonin in responding SBRT + IL-2 patients compared with non-responders for IL-2 monotherapy. The study was not designed to detect a difference in clinical outcome based on the site chosen for SBRT, but none of the patients who had liver as the only site treated with SBRT achieved control of melanoma. The mechanism for this lack of response may be related to fewer CD8 + T cells present in liver metastases and also decreased CD8 + T cell in tumor-infiltrating lymphocytes in non-liver sites in melanoma patients having liver metastases.^28^ The patients who had a pulmonary site chosen for SBRT had the highest proportion of response. Individual patients who had both a lung and liver site also achieved responses. This observation may be a consequence of the known poorer prognosis of patients who have melanoma liver metastases compared with melanoma lung metastases. It also suggests that the immune response after radiation in pulmonary sites may be different compared with radiation of hepatic sites. We are developing a clinical trial to investigate this hypothesis.

We acknowledge the small sample size, the influence of the crossover in interpreting response and survival and the lengthy time to meet accrual goals; however, the medical management of advanced melanoma changed dramatically from the time the study opened in late 2011 to the present with at least nine new medicines or regimens approved by the Food and Drug Administration. These changes in melanoma treatment altered the referral patterns to our cancer center of untreated patients compared with our original pilot study, which treated patients in the first line with SBRT + IL2.^24^

## Conclusions

We observed a modest trend toward higher response rates with SBRT + IL-2 and higher disease control rate in patients receiving dual therapy compared with IL-2 monotherapy. A larger study without a crossover group would be required to address overall survival in the current era of melanoma treatment in which multiple lines of therapy are commonly administered to patients with advanced melanoma. This study also illustrates that SBRT + IL-2 has activity in patients after progression on IL-2 monotherapy, CPI or BRAF-targeted therapy, the latter of which are the current standards of care for patients with advanced melanoma. Individuals with symptomatic metastatic sites amenable to treatment with high-dose per fraction radiation and physiological reserve sufficient to tolerate HD IL-2 should be considered for SBRT + IL-2 in the second or third line.

10.1136/jitc-2020-000773.supp6 Supplementary data
