# Supplementary figures and images for: Randomized phase II study of stereotactic body radiotherapy and interleukin-2 versus interleukin-2 in patients with metastatic melanoma
Source: J Immunother Cancer. 2020 May 27;8(1):e000773. doi: 10.1136/jitc-2020-000773 (PMC7259841; doi:10.1136/jitc-2020-000773)

Figure S3

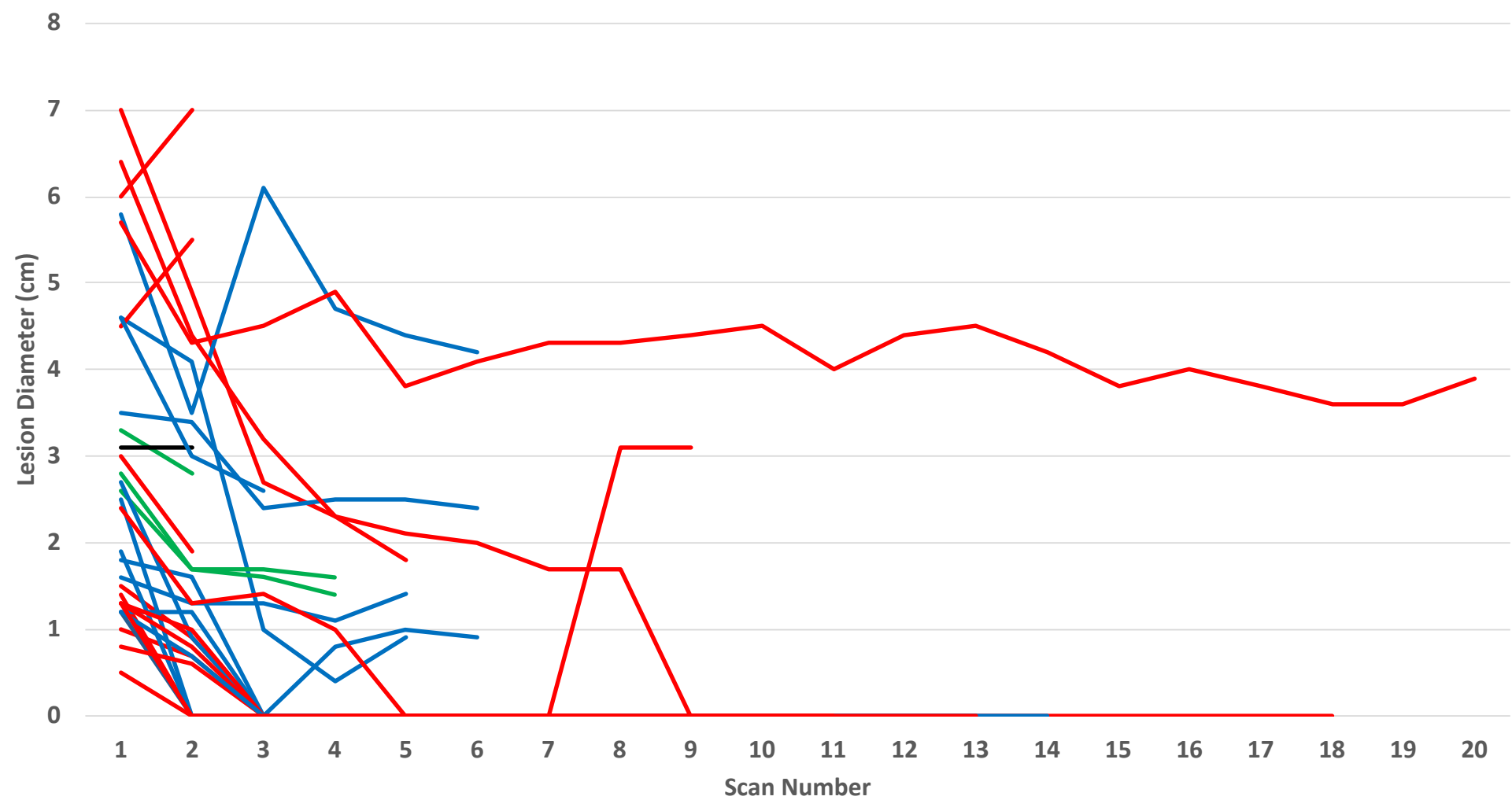

Supplement: Supplementary data [file jitc-2020-000773supp001.pdf]

Figure S1

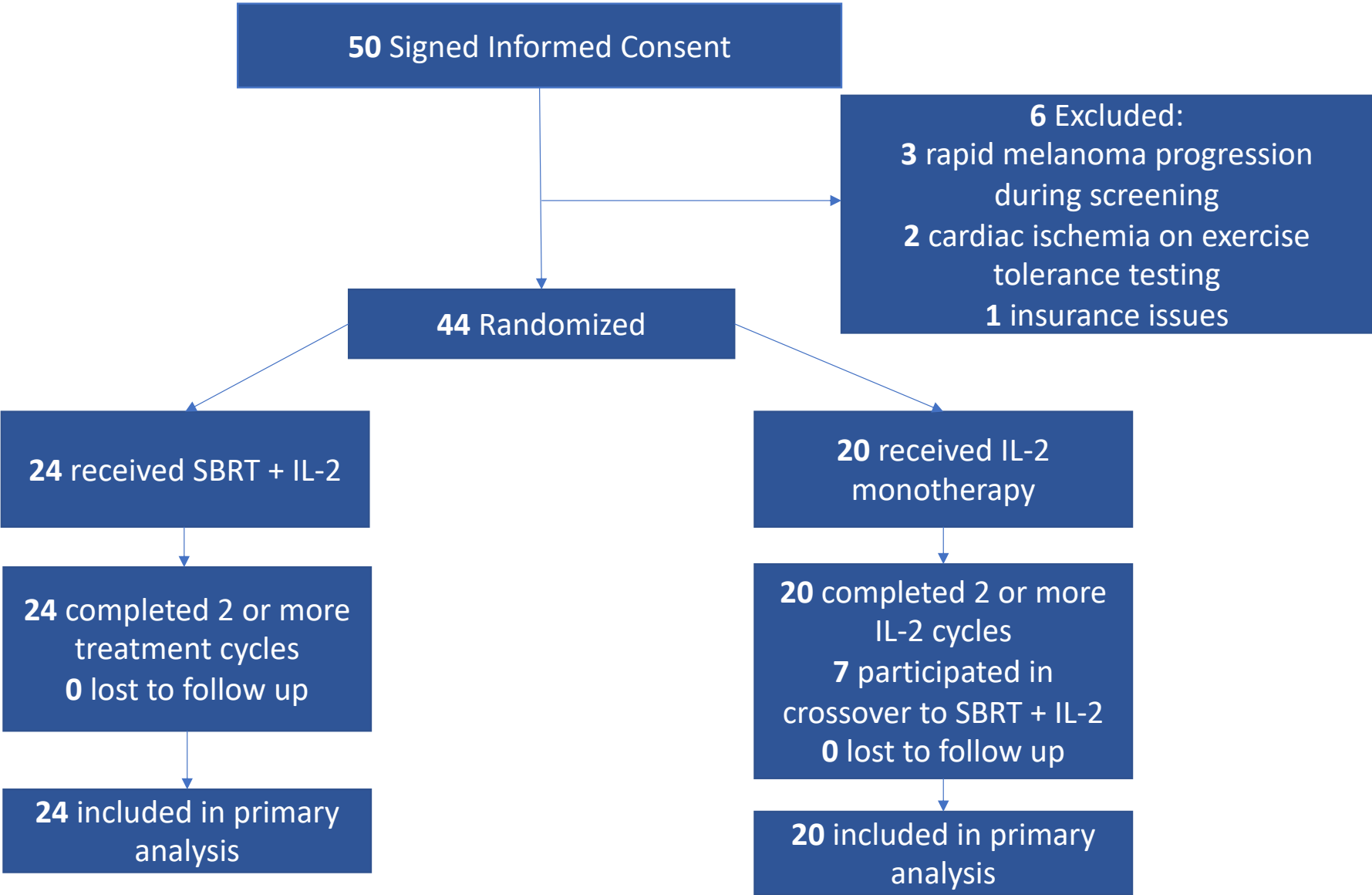

Supplement: Supplementary data [file jitc-2020-000773supp003.pdf]

Figure S2A

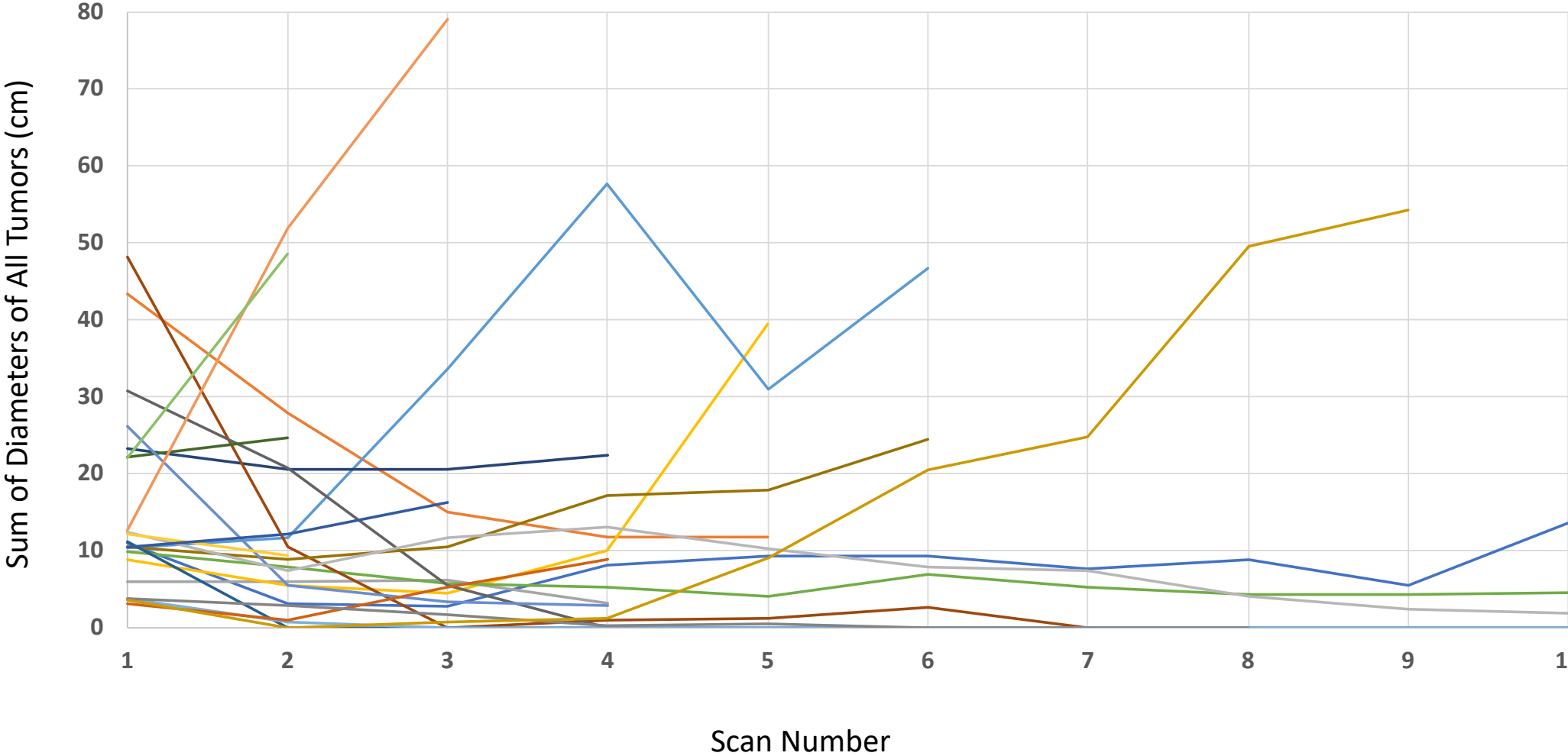

Supplement: Supplementary data [file jitc-2020-000773supp004.pdf]

Figure S2B

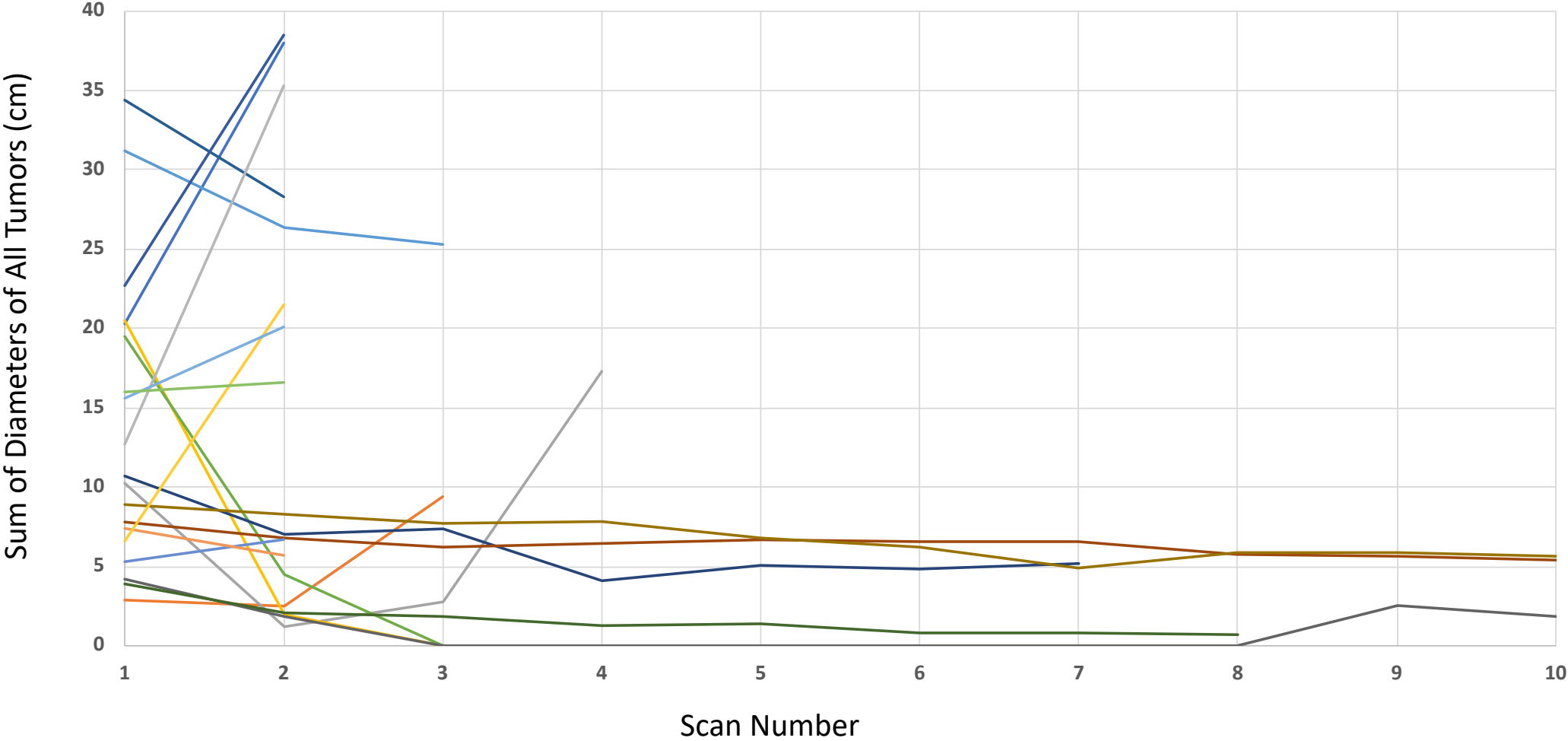

Supplement: Supplementary data [file jitc-2020-000773supp005.pdf]

Figure S4

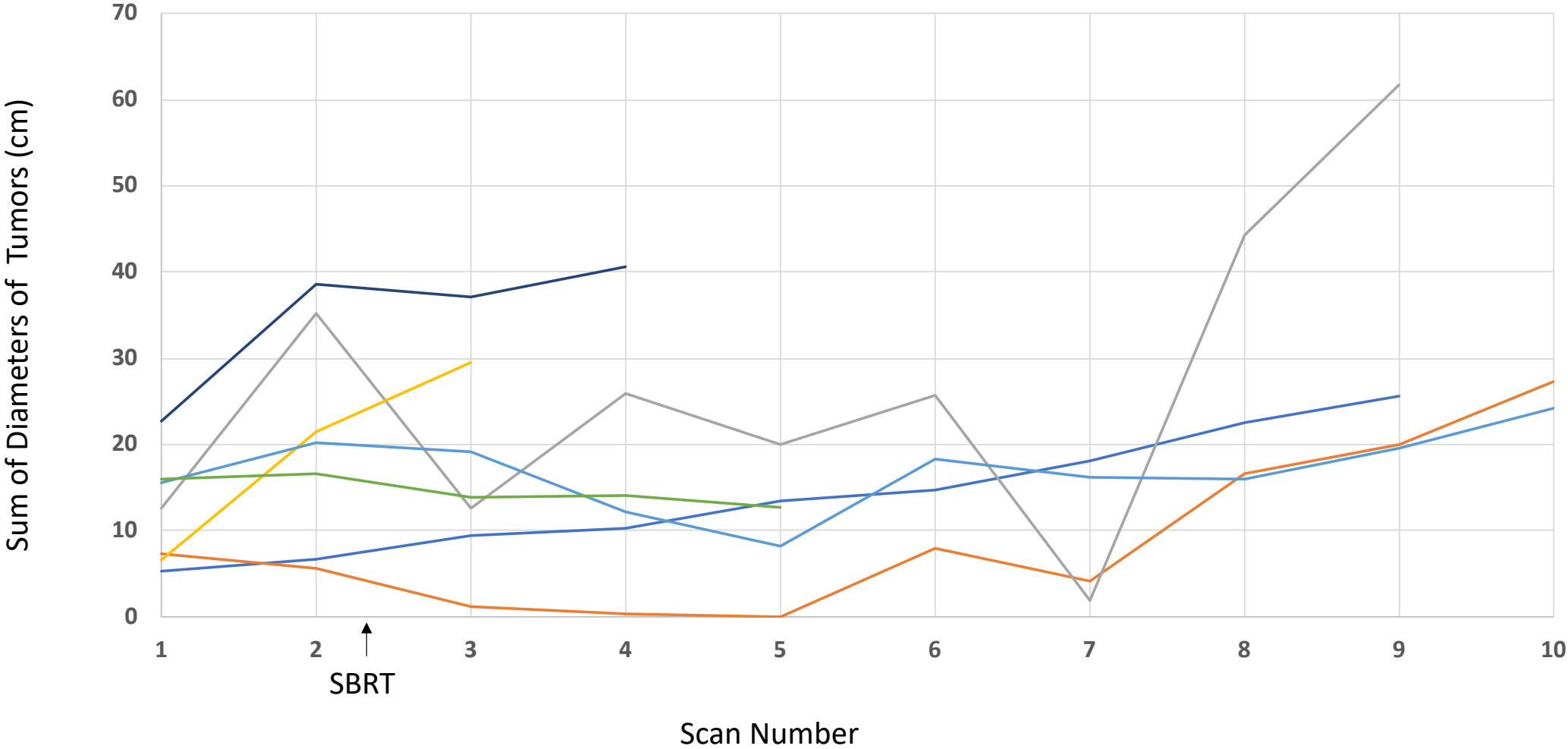

Supplement: Supplementary data [file jitc-2020-000773supp006.pdf]
