# Supplementary material for: Randomized phase II study of stereotactic body radiotherapy and interleukin-2 versus interleukin-2 in patients with metastatic melanoma
Source: J Immunother Cancer. 2020 May 27;8(1):e000773. doi: 10.1136/jitc-2020-000773 (PMC7259841; doi:10.1136/jitc-2020-000773)
Supplement: Supplementary data [file jitc-2020-000773supp002.pdf]

Supplementary Table 1 Univariate and Multivariable analysis examining the predictors of response (CR/PR/SD) using logistical regression.

	Univariate Analysis				Multivariable Analysis			
	OR*	Lower 95% CI	Upper 95% CI	p-value	OR*	Lower 95% CI	Upper 95% CI	p-value
Group								
IL-2 and crossover	Reference				Reference			
SBRT + IL-2	2	0.55	7.24	0.29	1.44	0.17	12.45	0.74
Age at diagnosis, years	1.02	0.97	1.08	0.37	1.05	0.96	1.15	0.29
LDH value with log_2 transform, U/L	0.5	0.23	1.10	0.08	0.44	0.12	1.65	0.22
Total Tumor Burden Baseline, cm	0.98	0.93	1.03	0.40				
Total Tumor Burden Nadir Increase, cm	0.85	0.77	0.94	0.002	0.88	0.8	0.96	0.01
Gender								
Female	Reference							
Male	1.6	0.37	6.92	0.53				
BRAF Status								
V600E	Reference							
WT	0.86	0.22	3.31	0.82				
Unknown	3	0.29	30.92	0.36				
*odds ratio of response (CR/PR/SD)								
